# The Influence of Art Expertise and Training on Emotion and Preference Ratings for Representational and Abstract Artworks

**DOI:** 10.1371/journal.pone.0134241

**Published:** 2015-08-05

**Authors:** Jorien van Paasschen, Francesca Bacci, David P. Melcher

**Affiliations:** 1 Center for Mind / Brain Sciences (CIMeC), University of Trento, Rovereto, Italy; 2 Museo d'Arte Moderna e Contemporanea di Trento e Rovereto (Mart), Rovereto, Italy; Cognitive Brain Research Unit, FINLAND

## Abstract

Across cultures and throughout recorded history, humans have produced visual art. This raises the question of why people report such an emotional response to artworks and find some works more beautiful or compelling than others. In the current study we investigated the interplay between art expertise, and emotional and preference judgments. Sixty participants (40 novices, 20 art experts) rated a set of 150 abstract artworks and portraits during two occasions: in a laboratory setting and in a museum. Before commencing their second session, half of the art novices received a brief training on stylistic and art historical aspects of abstract art and portraiture. Results showed that art experts rated the artworks higher than novices on aesthetic facets (beauty and wanting), but no group differences were observed on affective evaluations (valence and arousal). The training session made a small effect on ratings of preference compared to the non-trained group of novices. Overall, these findings are consistent with the idea that affective components of art appreciation are less driven by expertise and largely consistent across observers, while more cognitive aspects of aesthetic viewing depend on viewer characteristics such as art expertise.

## Introduction

People have created and appreciated visual artworks throughout history and across different cultures. Yet the question of what exactly it is about art that appeals to us remains largely unanswered. Experiencing an artwork is a complex phenomenon, likely involving a number of affective, motivational, and cognitive processes such as pleasure/reward, recognition memory, thinking, and reasoning [1,2]. Indeed, there is no consensus in the literature on which mechanisms underlie our perception of art or what exactly defines an aesthetic experience. Studies on the perception of art have traditionally explored contemplative appreciation of art such as judgments on the beauty of artworks [3–6] and to what extent artworks are “liked” or preferred [7–9]. The terms ‘beauty’ and ‘pleasure’ are often used interchangeably [10], presumably because a beautiful stimulus is considered rewarding and, hence, positive or pleasurable. However, beauty and valence are indeed two different dimensions: imagine a melancholic (negative valence) piece of classical cello music that brings a tear to your eye, yet you consider it beautiful. Silvia [11] pointed out that using an overarching measure like ‘preference’ can mask differences between separate components of art appreciation, such as interest and enjoyment—two measures that may diverge when investigated separately [12,13].

Some models of aesthetic viewing have emphasized the “beholder’s share” [14] in the aesthetic and emotional response to an artwork. Leder, Belke, Oeberst and Augustin [15], for example, have argued that knowledge of style and context of the artwork are vital to having an aesthetic experience. Aesthetic emotions are merely the by-product of progression through the cognitive stages in their model; understanding of style and content is thought to be self-rewarding, hence the more sophisticated the art expertise of the observer, the greater is the experienced self-reward. This is in line with an ‘effort after meaning’ theory which argues that part of what makes viewing art enjoyable is the ability to interpret the message and intentions of the artist [16].

Although expertise explains part of the appreciation of art, it does not account for emotions that may be triggered by visual features of the artwork itself. For example, it is known that people have emotional responses even to simple geometric features without a particular context [17,18]. Downward pointing triangles, and sharp angles in general, were experienced as especially threatening, while round shapes such as circles were deemed pleasant. Indeed, many authors have emphasised a special role for particular properties of artworks, such as composition, balance, and symmetry [19,20] that might be most likely to evoke an aesthetic response. Chatterjee has proposed a neuropsychological framework of visual aesthetics in which emotional experience plays a central role in aesthetic viewing [21]. He emphasises that for a visual artwork, like any visual object, visual characteristics engage fronto-parietal attention networks [22,23] resulting in heightened attention towards features related to high valence / arousal. Hence, early in the perceptual process, affective information can modulate how a given object is processed [24]. Importantly, in Chatterjee’s model the emotional experience is central to aesthetic viewing, although the role of art expertise and its interaction with aesthetic emotions need to be explored in more depth.

### Emotional aspects of aesthetic viewing

Chatterjee [21] and Scherer [25] have pointed out that emotions triggered by an artwork have no immediate utilitarian function and can be considered a matter of ‘wanting’ versus ‘liking’, or viewing with ‘disinterested’ interest. Scherer proposed that aesthetic emotions comprise being moved, being in a state of bliss, fascination, admiration, and so on. These emotions serve no physical goal (food, sex, etc) but they can trigger physical reactions such as moist eyes, or goose bumps [26]. It is not clear how or why the presence of aesthetic emotions should preclude the occurrence of ‘utilitarian’ emotions such as sadness or joy—these emotions can and do occur frequently without being action-oriented, for example when thinking about the loss of a loved one. Marković [1] also distinguished aesthetic emotions from other emotions, defining aesthetic emotions as generally pleasant “feelings of unity and exceptional relationship” with works of art (p.11), induced by the appraisal of physical characteristics of the object (structure, composition, etc.). However, he points out that evaluating the content of the artwork can induce a wider range of emotions not restricted to aesthetics, e.g. empathising with a character in a novel. Hence, pleasant or unpleasant emotions can exist alongside aesthetic emotions.

Previous theories on the emotional response to art have focused either on the intentional expression of a particular emotion by the artist [27–29] or on the experience of the observer [30–32]. In the former case, the hypothesis is that the artwork contains specific features that influence the perception of emotion. For visual art, this involves the manipulation of features such as shape, colour, texture, movement and depth [33–35].

Few studies have explored affective responses to artworks, let alone how levels of art expertise mediate this response. The one study that has focused on an emotional response in both art experts and novices [36] asked participants to rate the quality of emotion evoked by an artwork on a positive/negative dimension. It is not clear whether the authors referred to the felt emotion the artwork evoked *within* the participant, or to the perceived emotion that the artwork conveyed (although this may be a language issue). On average, the experts rated the artworks as more positive than did the novices, reflecting the novices’ more negative ratings for more abstract artworks. However, this study included a limited set of paintings and, art expertise not being the main focus of the study, the observed differences between the groups were not further discussed.

In their study on the effect of different types of titles on aesthetic experience, Leder, Carbon and Ripsas [9] included a question on how much participants ‘were affected’ by a particular artwork. Participants were all art novices with no particular background in art. Interestingly, providing a descriptive title led to *decreased* affective ratings for both abstract and representational (depicting recognizable real-life scenes, objects, and / or people) artworks. The authors hence argued that the presence of a title made the work less aesthetically preferable and interesting. Alternatively, it is possible that providing a descriptive title may have prompted participants to approach the artwork in a more cognitive manner, perhaps focusing attention on specific objects or themes within the painting, rather than letting the artwork speak for itself.

In a different study comparing representative and indeterminate (paintings that suggest the presence of, but do not actually contain, real-world objects) artworks, participants who had no training in art also indicated how much the artworks affected them [37]. No differences in affective ratings were found between the two types of paintings, suggesting that an affective response to art may be independent of a meaningful content.

Some researchers have highlighted the concept of felt versus perceived emotion [38]. Gabrielsson distinguishes between a felt emotion, which is someone’s actual intrinsic emotional response, and perceiving an emotional expression in art or music—for example, being able to identify that a piece of music is cheerful without necessarily being affected by it oneself. In addition, even when participants rate a specific emotional question (e.g. “how positive / negative do you think this artwork is?”), the wording of the question may not enable the researcher to decipher whether the participant *felt* positive upon viewing the artwork, or whether the participant *perceived* the artwork as expressing joy.

Our group has previously shown that observers with no training in art awarded highly consistent ratings of valence and arousal to abstract works of art [39,40]. In addition, in a computational vision study, it was possible to train a classifier to correctly predict human emotion judgments for abstract artworks based on basic visual features [41]. These findings are in line with the idea that valence and arousal judgments are formed, at least in part, on the basis of visual features of the artworks (e.g. line, shape, colour), consistent with Chatterjee’s model of aesthetic perception described earlier. The focus on valence and arousal stems from the core affect theory of emotion [16] that views affect as the experience of neurophysiological states along a two-dimensional scale consisting of valence (pleasure / displeasure) and arousal (calm / activated). A similar view of emotion seen along several dimensions has been proposed by Barrett [42] and Bradley and Lang [43,44], who devised the well-known International Affective Picture System (IAPS) [45].

In conclusion, affective responses to art have been somewhat understudied, and little is known about the relation between level of art expertise and aesthetic emotion. It is important to distinguish between ‘felt’ and ‘perceived’ emotion when asking participants about their affective responses to an artwork. In the current study we studied aesthetic as well as affective aspects of art appreciation in both art experts and art novices. We asked participants to evaluate an artwork on valence, arousal, beauty, and “wanting”, focusing explicitly on how the artwork made the participants feel.

### The role of art expertise in art appreciation

Although a judgement of beauty may indeed be influenced by an emotional response, it is also thought to be heavily influenced by artistic style, art-historic knowledge and so on (see [15]). Thus, beauty and liking judgments would be more likely to reflect individual differences in expertise. In one study, for example, participants with varying levels of art knowledge were asked how much they liked a set of abstract artworks [7]. For half the artworks, stylistic information was provided, while the others were viewed without any extra information. Results showed that participants with low art expertise preferred paintings for which stylistic information was provided compared to those without any information, while participants with a high art expertise showed the opposite pattern. With regard to the naïve observers, the authors suggested that stylistic knowledge increased a rewarding feeling when participants viewed the artwork. To the experts, this information may have seemed trivial, or was a repetition of what they already knew.

A different study looked at the effects of manipulating representative artworks (digitally creating black-and-white, and degraded—more abstract—versions) on overall preference of the artwork in a group of art novices, ‘relative’ experts (graphic design students with applied knowledge of visual arts), and art experts [8]. Art novices and graphic design students rated the representative works significantly higher than the abstract works, and also preferred coloured paintings to black and white versions. The experts, however, did not show this difference in ratings—arguably because experts appreciate novelty and originality in an artwork.

Pihko and colleagues [36] asked art experts and novices for an aesthetic evaluation (“Is this a good work of art?”) and an emotional judgement (“What is the quality of emotion evoked by this painting?”) of artworks varying in abstraction level. Both questions were rated on a five-point Likert scale. For the emotional question, the scale used was from ‘very positive’ to ‘very negative’. All participants completed one rating session with and one without background information on the artworks. Because one of the main aims of this study was to investigate the relationship between expertise and abstraction level of the paintings, the relation between background information and aesthetic and affective judgements was only briefly touched upon. Ratings from experts and novices on aesthetic evaluations were not compared directly to one another. Aesthetic ratings were higher overall (i.e. irrespective of group) for the session where background information was provided, whereas this made no difference to affective ratings. Although these findings are not further discussed, it is intriguing and relevant to the current study that changes in ratings only occurred for aesthetic but not for affective evaluations.

In sum, previous studies comparing preference ratings between art experts and novices have found important differences between these two groups. Art novices have been reported to prefer representative to abstract art, and like art more when stylistic information is present. Art experts do not show any particular preference for abstract or representative art, and preferred to view artworks without stylistic information.

### The role of context in aesthetic perception

While most studies on art perception take place in a laboratory and use reproductions of artworks presented on a computer screen, most artworks have been created in order to be experienced as real, three-dimensional objects in museums, art galleries, private collections, churches, and so on. Some models of art perception emphasise the role of the viewer’s expectations, for example by pointing out that it is important to pre-classify the object as art [15,46]. The idea that aesthetic appreciation is modulated by context is supported by a study in which participants were asked how much digitalised images of 200 abstract artworks appealed to them [47]. Of note, although all stimuli were images of real artworks, half were labelled to be computer-generated images while the other half was labelled as artworks belonging to a museum. Participants (who were art novices) found the images labelled as artworks to be more appealing than the supposed computer-generated images. This suggests that contextual knowledge about an object can affect the appeal of that object.

A museum, in particular, is considered to be the type of context that typically induces an aesthetic way of viewing a stimulus. This raises the question of whether such conditions are really replicated when viewing art on a computer screen in a lab. Historically, most artworks have not been flat, digital images of a fixed screen size, but instead artists made specific choices about the size, shape and surface texture. A large canvas with thick and uneven brushstrokes may have a different visual effect on the viewer compared to a small watercolour, but much of this difference is lost in a lab setting. Perhaps the only way to test the role of “being there” is to directly compare the responses of participants to the same group of artworks in a museum and a laboratory. A few studies have done this, typically with only one or a few specific works [48,49]. Locher and colleagues directly compared ratings on a variety of measures for artworks seen in a museum and the same works presented as a digital image on a screen [48]. While there were no differences in ratings on physical characteristics of the artworks (e.g. judging complexity, symmetry, crowdedness), artworks viewed in the museum were considered more pleasant and more interesting compared to the reproductions viewed on a monitor.

### Familiarity and aesthetic viewing

The mere exposure effect refers to increased preference when confronted with a stimulus one has encountered before [50]. This idea has been further elaborated into a two-factor model [51] that holds that stimulus habituation initially produces positive affect as the viewer learns that the stimulus does not present a threat. Overexposure, however, results in boredom and thus leads to depreciation of the stimulus. Stang [52], in a series of experiments, showed that repeated exposure to a stimulus involves learning, which is intrinsically rewarding. Hence, exploring a stimulus renders positive reinforcement. Simple stimuli are learned faster, and boredom may thus set in more quickly compared to complex stimuli. Interestingly, Stang [53] found that the mere exposure effect appears to be weaker for paintings compared to other types of visual stimuli. However, he pointed out that the majority of studies including paintings did not incorporate a period of delay between the first and second presentation, something that is considered important to achieve increased preference at the time of the second viewing. A meta-analysis of studies concerning the mere exposure effect also found that this effect is somewhat weaker for paintings and line drawings [54]. On the contrary, a different study found a positive correlation between familiarity and preference ratings for a set of Van Gogh paintings [55]. This relationship was weakened with longer viewing time, and was also reduced when participants were told that the paintings were ‘fakes’. Hence, the relationship between familiarity and aesthetic preference seems intricate, and findings from previous studies are equivocal.

### The current study

We investigated the roles of observer experience and training, familiarity, and the physical context in which the artwork is viewed, on judgments of emotion, beauty and preference for abstract artworks and representational artworks (in this case, portraits). We took advantage of the occasion of the ‘La Magnifica Ossessione’ exhibition held at the Contemporary and Modern Art Museum of Trento and Rovereto (Mart), in which more than 2000 works were displayed in separate, themed rooms. Thus, we were able to present participants with the same artworks, 50 portraits and 100 abstract artworks, in the lab and in the museum space. Importantly, in the exhibition there were no labels next to the artworks indicating the title or artist. Otherwise, such information might have influenced observers [9,31,56].

In the present study we investigated the effect of art expertise on art appreciation using both basic emotion judgments (valence and arousal) and more aesthetic judgments of beauty and liking. In order to study the role of art expertise, we tested three groups of participants. One group was highly trained in art and art history and regularly visited museums. The other two groups had little or no background in art but one of the groups received a brief training session on abstract art and on portraiture. Many museums have specific education departments whose remit is to provide supporting context and information with the theory that such experiences can impact the experience of the museum visitors. Thus, we were able to explore empirically the effects of a brief training in artistic aspects of the artworks on art appreciation in non-expert observers.

Based on previous studies, we predicted that art expertise would affect aesthetic appreciation mainly with respect to beauty and liking judgments. Furthermore, based on studies showing a link between emotional judgments and low-level attributes of artworks (colour, shape, composition) we anticipated that affective judgments with respect to arousal and valence would be similar for art experts and novices alike. Secondly, we hypothesised that artworks viewed in the museum would be rated higher overall compared to their digital counterparts viewed in the lab. Finally, we expected that during the museum session, artworks seen previously in the laboratory session would be preferred to artworks seen for the first time in the museum. Based on the processing fluency account of the mere exposure effect [10], familiar portraits were thought to receive higher ratings compared to familiar abstract artworks as we considered portraits easier to process.

## Methods

### Participants

Twenty art experts—visual artists, art teachers / students, museum workers—and 40 participants who had no particular background in art took part in the experiment. Participants were tested over two sessions: the first session always took place in the laboratory, while the second session was held in a museum four to seven days after the first session. At the start of the second session, the naïve group was divided into a ‘Training’ (TR; n = 20) and a ‘No Training’ (NT; n = 20) group. The TR group received a training specific to the artistic style and historical period of the paintings viewed. Demographic data about the groups are provided in Table 1.

**Table 1:** Demographic characteristics of participants.

Group	Gender	Age	Years of education	Art expertise score (max. 46)
*No training*	7 male / 13 female	24.9 ± 6.7	13.1 ± 1.9	4.7 ± 3.0*
*Training*	6 male / 14 female	22.8 ± 4.7*	13.5 ± 1.5	6.9 ± 4.7*
*Expert*	5 male / 15 female	28.5 ± 7.9	13.8 ± 2.0	27.2 ± 5.5

The art expertise score refers to a questionnaire adapted from the Assessment of Art Attributes (see Measures section) in which participants indicated, among others, the number of hours per week spent on creating visual art, number of museum visits per year, and so on. Scores are presented as mean ± standard deviation.

* significantly lower compared to the Expert group at *p*<.005.

Participants were students of the University of Trento, members of the local community, artists, and art teachers. Participants were recruited through advertisements on the internet and through emails sent out to art experts registered with the Contemporary and Modern Art Museum of Trento and Rovereto (Mart). All gave written informed consent prior to their participation. To ensure comparable ages in all groups, only people under 45 years of age were invited to participate. Exclusion criteria were visual impairments that could not be corrected by glasses / contact lenses, age over 45 years, and having already visited the exhibition at the Mart from which we drew our stimuli. Participants received a monetary reimbursement for their participation. The experimental procedures were approved by the ethical committee of the University of Trento, Italy, and adhered to the principles set out in the Declaration of Helsinki.

### Materials

Stimuli were 100 abstract artworks and 50 portraits that were all part of the exhibition ‘La Magnifica Ossessione’ held at the Mart. We used artworks displayed together in six different rooms in the exhibition. We included abstract artworks from the art streams Abstract Expressionism and Geometric Abstraction [see 34,35], with the restriction that they were completely abstract and contained no recognisable objects, figures, letters, or numbers. To allow for a comparison with representational art, we also included portraits. The portraits were chosen on the basis that the face was clearly visible. Most portraits displayed only the head; some depicted the individual from the waist up. Portraits including multiple people and / or nudes with clearly visible bodies were excluded from testing.

The artworks were divided into two stimulus sets (henceforth referred to as set 1 and set 2) in a pseudo-random manner based on their size, ensuring that artworks from each exhibition room were divided roughly equally over the two sets and that each set contained similarly sized artworks. There were 50 abstract artworks and 25 portraits in each set. In the first session participants only viewed one set, while in the second (museum) session they viewed all 150 stimuli. This was done to explore possible effects of familiarity—in the museum session half of the artworks was novel to participants while the other half had previously been viewed in the first session and was thus considered to some degree familiar.

For the first part of the experiment, which took part in the laboratory, we used professionally made digital images of the artworks taken from the museum archive. The largest dimension of each image was resized to 500 pixels while the other dimension was adjusted relative to the first, so that the original dimensions were preserved. The artworks were presented on a Toshiba Satellite Pro L500-1VZ laptop using NBS Presentation software.

### Measures

To assess art experience, participants completed a questionnaire that was adapted from the Assessment of Art Attributes (AAA) [57]. The questionnaire included multiple choice questions on art classes attended, museums / galleries visited per year, and time spent per week on creating visual art, with possible answers ranging from ‘zero’ to ‘six or more’.

### Experimental task

Participants were asked to rate the artworks on four dimensions (one rating per dimension): Valence, Arousal, Beauty, and Liking. Ratings were done on a 7-point Likert scale. In the laboratory session, participants used the numeric buttons 1 to 7 on the keyboard. In the museum session participants were given a paper questionnaire on which they circled their answers. The Valence dimension (scale used: sad / happy; Italian wording: triste / felice) was described to participants as the extent to which the artwork made them feel happy, pleased, satisfied, contented, hopeful, or, on the opposite end, unhappy, annoyed, unsatisfied, melancholic, despaired, bored. We described the Arousal dimension (scale used: calm / exciting; Italian wording: calmo / eccitato) as the extent to which viewing the artwork made participants feel stimulated, excited, frenzied, jittery, wide-awake, aroused, or instead relaxed, calm, sluggish, dull, sleepy, un-aroused (the descriptions for Valence and Arousal were taken from the IAPS manual, see [45]). On the Beauty dimension (scale used: ugly / beautiful; Italian wording: brutto / bello), participants specified to what extent they thought the artwork was on the one hand beautiful, stunning, delightful, excellent, or instead, ugly, unattractive, disagreeable, revolting. Finally, on the Liking dimension (scale used: I don’t like it / I like it; Italian wording: non mi piace / mi piace), participants were asked whether they would hang this painting in their own home (irrespective of practical issues such as space or value). The scale ranged from “loving to hang the painting in their home, feeling this was awesome and willing to take it right now if this was possible”, to “disliking it and not even considering hanging it in their own home”.

### Training

Half of the naïve participants were randomly allocated to receive the training session just prior to the start of their second (museum) session. The training comprised a 30-minute video clip presented on a laptop set up in a booth in the reception area of the museum. The video contained general background information about the artistic style, the artists, and art-historical background relating to the artworks in the exhibition. As such, the training provided participants with a theoretical framework and historical perspective in which to place the artworks viewed during the exhibition, while at the same time familiarizing them with the artistic style used in these artworks. The video contained approximately 15 minutes on portraits and 15 minutes on abstract art. None of the artworks tested was shown in the training videos.

### Procedure

The experiment consisted of two separate sessions: a laboratory session (Session I) and a session at the Mart (Session II). As described above, participants saw only one half of the artworks in the lab session and all 150 artworks in the museum setting. Where possible, sessions were planned four to seven days apart.

**Fig 1:**
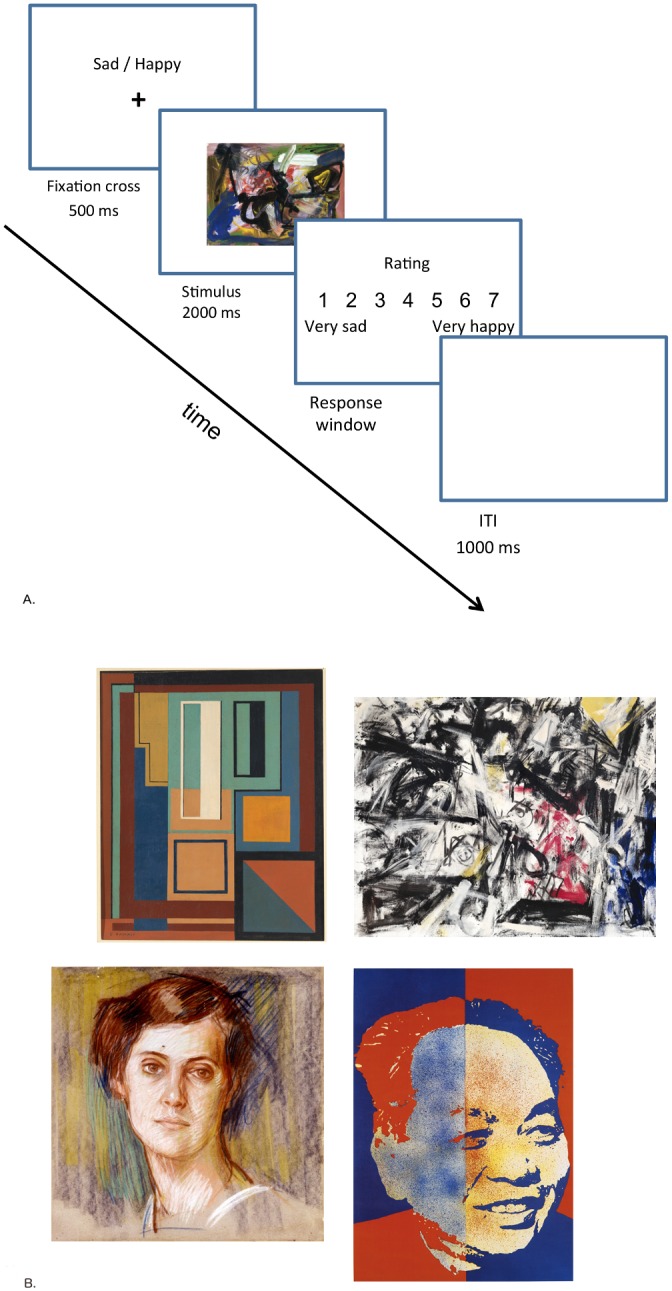
Examples of paradigm and stimulus material used in the laboratory session. (A) The experimental paradigm used in session I (laboratory session). (B) Examples of the stimuli. The top row shows examples of Geometric Abstraction (left) and Abstract Expressionism (right) artworks, while the bottom row shows sample portraits.

The first session was always held in the laboratory, where participants were asked to rate a set of paintings (i.e. half of the complete stimulus set) presented on a computer screen on the four different dimensions described in the previous section. Participants were randomly assigned to rate artworks from either set 1 or set 2, as described above. Participants completed the art expertise questionnaire and a short practice with a different set of stimuli before engaging in the actual task. Each trial started with a fixation cross, presented for 500 ms in the centre of the screen. Above the fixation cross we provided a cue with regard to which type of dimension the participant would be rating the painting on, for example ‘happy / sad’ served as a cue for the Valence dimension. The artwork was then presented for 2000 ms, during which time it was not possible to make a response. Next, a rating screen appeared, stating the dimension and a scale from 1 to 7 with a written description provided for each extreme of the scale. The experimental paradigm is illustrated in Fig 1.

The questions and paintings were presented in random order, so that each painting was seen four times in total over the course of the testing session. The rating was self-paced, but participants were encouraged to respond as quickly as possible and to not ‘overthink’ their reaction to the artwork. The laboratory session lasted approximately 45 minutes.

Following the first session, half of the naïve participants were randomly allocated to the training condition. They were informed of this upon arrival at the museum, at the start of their second session. Participants then made their way through the museum, visiting the six rooms that displayed the artworks from the experiment.

The second session always took place at the museum, typically 4–7 days after the first session. In the museum the artworks from both stimulus sets (the previously seen set and a new set for each participant) were on display (among many more) in a large exhibition. To discourage participants from lingering in the museum during the experiment, we asked them to go straight to the rooms exhibiting the artworks they needed to rate. After the experiment was completed participants had the opportunity to visit the full exhibition free of charge.

**Fig 2:**
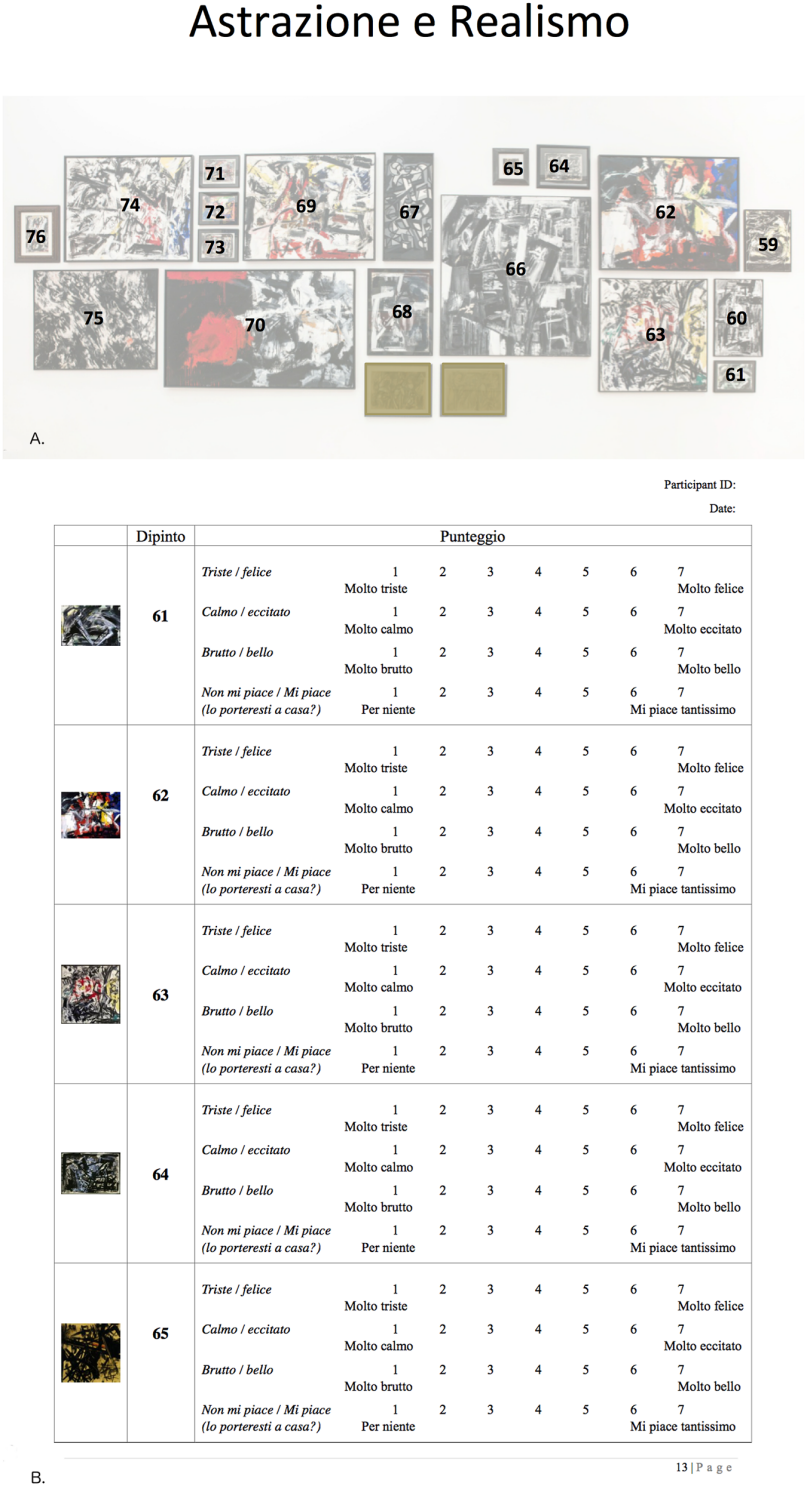
Example of materials used in the museum session. (A) An example of a hand-out displaying one of the exhibition rooms in the museum with artworks included in our stimulus set, to aid participants in identifying the correct paintings and their corresponding numbers on the rating sheet. (B) Part of the rating sheet used in the museum session.

For practical reasons, it was not possible in the museum session to randomise the order in which the paintings were viewed. To minimise order effects, we used two different routes (clockwise and anticlockwise) via which participants made their way through the exhibition. Participants were randomly asked to follow either the clockwise or the anticlockwise route, and were provided with a map of the exhibition on which the six relevant rooms were highlighted. They performed a paper-and-pencil rating of all the artworks in the experiment (i.e. 100 abstract artworks and 50 portraits) on the four dimensions, which were printed to the right of a small image of the artwork on the rating sheet. We provided photographs of the relevant exhibition walls on which the paintings were numbered in a manner that corresponded with the rating sheet (Fig 2). The rating task was self-paced and took on average 1.5 to 2 hours to complete.

### Planned data analysis

Because the four evaluative questions (valence, arousal, beauty, and wanting/liking) are not independent of one another, and their scales not comparable, we analysed ratings for each question separately. The data analysis entails three parts.

Firstly, we investigated effects of art expertise on ratings of the four evaluative questions. This was done using only data collected at the first (baseline) session in the laboratory. To explore the effect of art expertise at baseline, we entered data for each question into a 2 by 2 repeated measures analysis of variance, using Group (Naïve, Expert) as a between-subjects factor and Art Type (Portrait, Abstract) as a within-subjects factor. Please note that the naïve group was not divided into a training and non-training group until the start of the second session at the museum, hence in the baseline session at the laboratory there were only two groups. We report significant main effects and interactions at a threshold of *p*<.05.

Secondly, we looked at the effects of providing a short training session. To this end, we compared ratings on the four questions collected during the first session with ratings for new paintings from the museum session. Again, ratings were analysed separately for each of the four questions. We compared ratings from the baseline session (Laboratory; all paintings viewed for the first time) with ratings for paintings that were viewed for the first time in the museum (Museum). Ratings for familiar artworks (seen in both the lab and consequently in the museum) were analysed separately. Data were entered into a 3 by 2 by 2 repeated measures analysis of variance, using Group (Non-training, Training, Expert) as a between-subjects factor, and Session (Laboratory, Museum) and Art Type (Portrait, Abstract) as within-subjects factors. As before, we report significant main effects and interactions at a threshold of *p*<.05. Follow-up independent and paired samples *t*-tests were carried out to explore the nature of those results that reached statistical significance. These were Bonferroni corrected for multiple comparisons.

## Results

### Demographic characteristics

As expected, the Expert group scored much higher on the art expertise questionnaire compared to the Non-training (NT) and the Training (TR) group (*t*(38) = 12.545, *p*<.001) (see Table 1 for means and standard deviations). Unexpectedly, the TR group was significantly younger (mean age 22.8 years) than the Expert group (mean age 28.5 years) (*t*(30.832) = 2.764, *p* = .010; degrees of freedom are corrected because Levene’s Test for equality of variances was violated), most likely because the naïve participants were predominantly students within a narrow age range. No differences were found in educational level between the groups (*F*(2,57) = .643, *p* = .529, *n*.*s*.).

### Effects of art expertise

To investigate how art expertise affects evaluations of valence, arousal, beauty, and liking, we used ratings from the baseline session at the laboratory. Because allocation of participants to the training or non-training group took place *after* completion of the first session, we analysed results from the first session using only two groups: the Naïve group (n = 40) and the Expert group (n = 20). Scores were entered into a 2 by 2 repeated measures analysis of variance with Group (Naïve, Expert) as a between subjects factor and Art Type (Abstract, Portrait) as a within-subjects factor. Ratings were analysed separately for each different question.

#### Valence

We did not observe any significant main effects or interactions in ratings of Valence.

#### Arousal

We found a significant main effect of Art Type (*F*(1,58) = 61.038, *p*<.001, η = .513). This effect was caused by portraits yielding more calm / unaroused ratings (mean 3.5±0.7) compared to abstract artworks (mean 4.1±.7), irrespective of Group.

#### Beauty

There was a significant main effect of Group (*F*(1,58) = 12.127, *p* = .001, η = .173), with the Experts rating all artworks as more beautiful (mean 4.3±0.8) compared to the Naïve group (mean 3.8±0.8).

#### Liking

Again, we observed a significant main effect of Group (*F*(1,58) = 12.104, *p* = .001, η = .173). This group difference was caused by the Experts liking the artworks overall (mean 4.1±0.9) more than the Naïve participants (mean 3.6±0.9).

**Fig 3:**
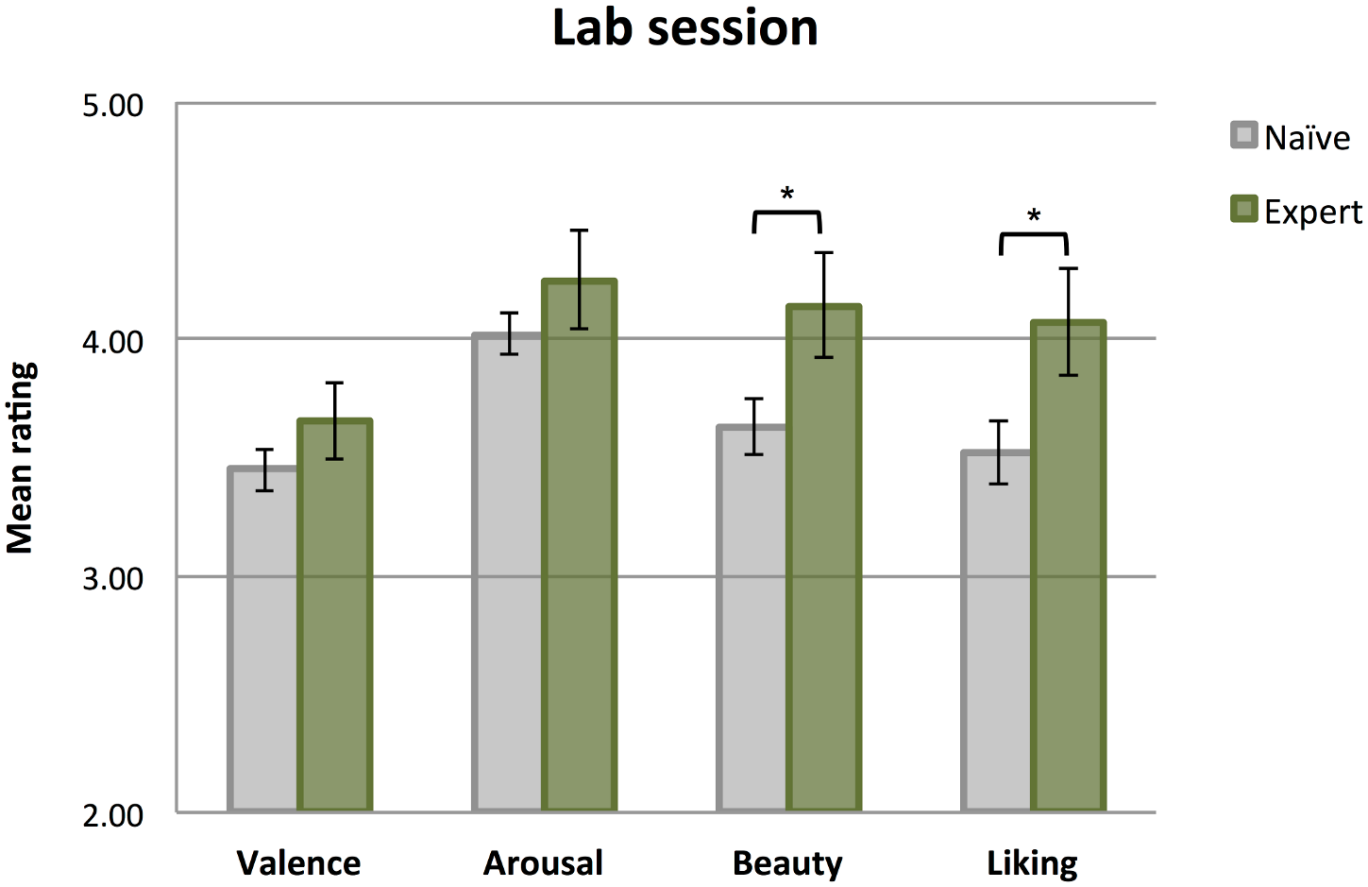
Mean ratings from naïve and expert participants in the laboratory session. * significantly different at *p*<.05 (Bonferroni corrected for multiple comparisons). Error bars represent standard error of the mean.

In sum, at the baseline session in the laboratory we found effects of art expertise on Beauty and Liking ratings, but not on dimensions of Valence and Arousal. Art experts rated the artworks as more beautiful and more likable personally compared to art novices. Irrespective of expertise, portraits were rated by both art experts and novices as more calm / non-arousing compared to abstract artworks. These results are illustrated in Fig 3.

One of the main interests of this study was to explore whether there is a relation between art expertise and art appreciation. We predicted that such a relationship would exist for more cognitively-influenced aspects of art appreciation (such as judging its beauty) but not when it comes to emotional experience of the artwork. This assumption is especially relevant for abstract artworks, where no recognisable objects are depicted that could trigger semantic associations. To investigate this idea, we correlated all participants’ expertise scores with their ratings on the four different dimensions. We used only the ratings from the baseline session, before participants had received any training.

**Fig 4:**
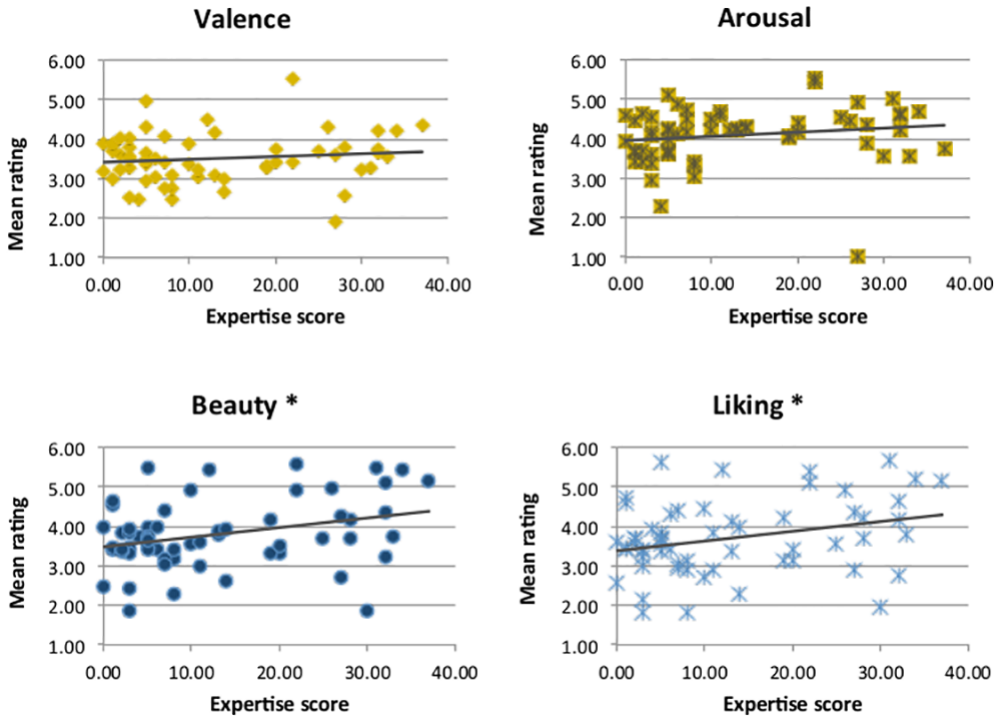
Correlation between participants’ rating on each dimension and their level of art expertise. * correlation significant at *p*<.05.

As predicted, expertise scores correlated positively with ratings on Beauty (*r* = .434, *p* = .001) and Liking (*r* = .444, *p*<.001) but not with the pure ‘emotional’ dimensions Valence (*r* = .184, *p* = .159) and Arousal (*r* = .235, *p* = .071). These results are depicted in Fig 4.

### Effects of training and context

To explore effects of training, as well as effects of art expertise and familiarity / setting, ratings from all three groups and both testing sessions were entered into a 3 by 3 by 2 repeated measures analysis of variance using Group (NT, TR, Expert) as a between-subjects factor and Session (Laboratory, Museum), and Art type (Abstract, Portraits) as within-subjects factors. Please note that only ratings for artworks seen for the first time in the museum are included in this analysis. Ratings for artworks that had already been seen in the first (laboratory) session are analysed separately in the next section on familiarity. We expected any effects of training to be characterised by a Group by Session interaction, with the Training group rating differently following training, but the Non-training and Expert groups behaving more or less the same across sessions.

#### Valence

We found no significant main effects or interactions with regard to Valence ratings.

#### Arousal

The analysis revealed a significant main effect of Art Type (*F*(1,57) = 95.556, *p*<.001, η = .626). This was driven by portraits being rated as more calm (mean 3.4±0.7) than abstract artworks (mean 4.0±0.7) irrespective of Group or Session.

#### Beauty

Results showed significant main effects of Art Type (*F*(1,57) = 6.707, *p* = .012, η = .105), Session (*F*(1,57) = 5.518, *p* = .022, η = .088), and Group (*F*(2,57) = 3.780, *p* = .029, η = .117). Portraits were rated as more beautiful (mean 4.0±0.7) than abstract artworks (mean 3.7±0.8). Interestingly, artworks viewed in digital format on a computer screen—irrespective of type—were rated as more beautiful (mean 4.0±0.6) than those viewed in their original format in the museum (mean 3.8±0.7). Finally, a follow-up independent samples *t*-test showed that the main effect of Group was driven by the Experts rating the artworks overall as significantly more beautiful (mean 4.1±0.6) than the Non-training group (mean 3.6±0.5) (*t*(38) = 2.782, *p* = .008), whereas no significant differences existed between the Experts and the Training group (*t*(38) = 1.228, *p* = .227) or between the Training and the Non-training group (*t*(38) = 1.526, *p* = .135) (all tests Bonferroni corrected for multiple comparisons).

#### Liking

We found a significant main effect of Session (*F*(1,57) = 18.179, *p*<.001, η = .458) as well as a main effect of Group (*F*(= 2,57) = 3.730, *p* = .030, η = .116). As with the Beauty ratings, participants rated the artworks seen in digital format in the laboratory as more preferred (mean 3.8±0.6) compared to artworks viewed in the museum (mean 3.2±0.8). Using follow-up independent samples *t*-tests, we found that the main effect of Group was caused by the Experts rating the artworks as more liked overall (mean 3.8±0.7) compared to the Non-training group (mean 3.2±0.6) (*t*(38) = 2.771, *p* = .009), whereas no statistically significant differences existed between the Experts and the Training group (*t*(38) = 1.222, *p* = .229), or between the Training group and the Non-training group (*t*(38) = 1.509, *p* = .140).

**Fig 5:**
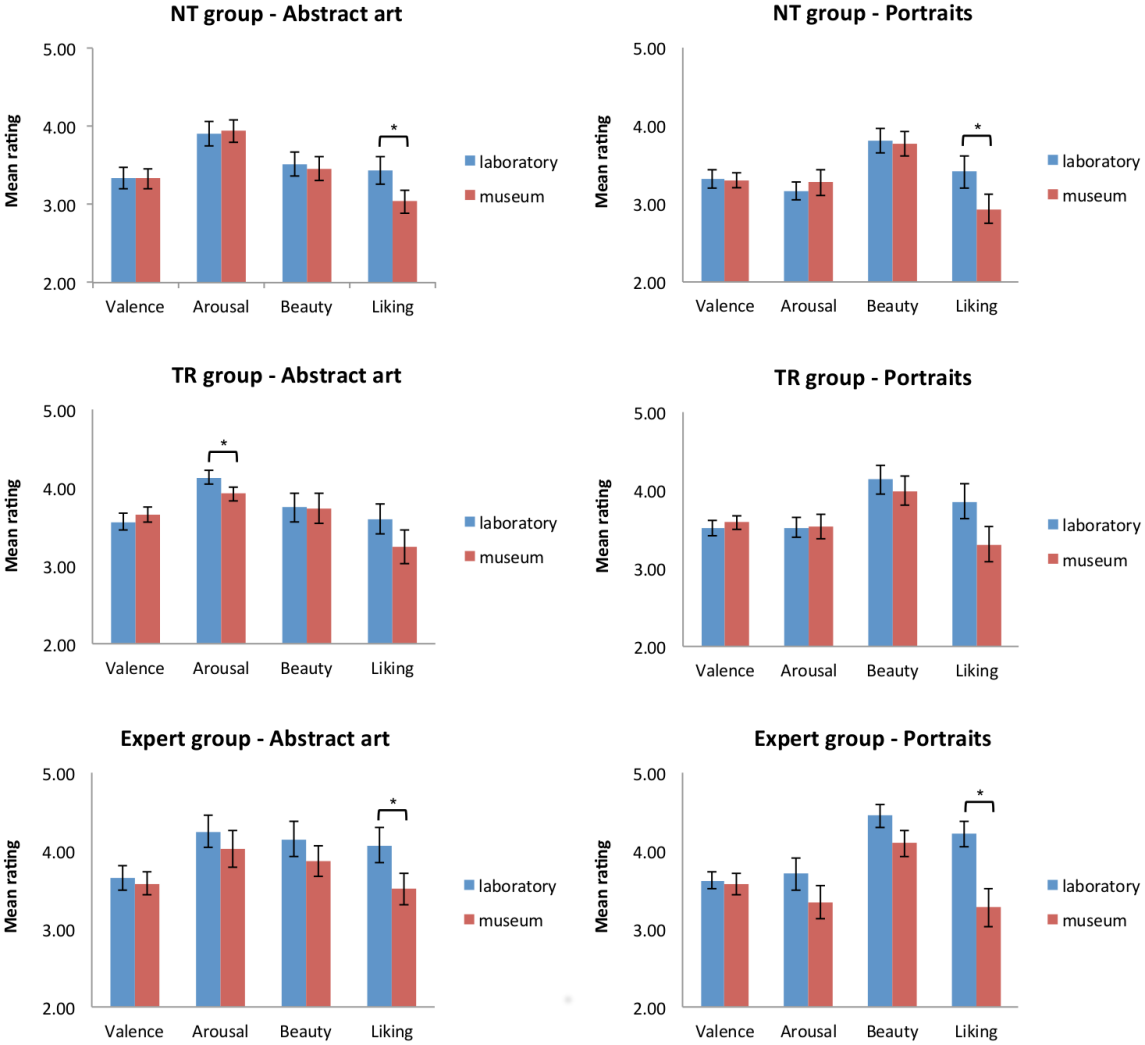
Effects of training and viewing context on ratings. Ratings for abstract art (graphs on left) and portraits (graphs on right) for all dimensions from all three groups at the laboratory session (blue bars) and the museum session (red bars). * significantly different from each other at *p*<.05 (Bonferroni corrected for multiple comparisons). NT = No Training; TR = Training. Error bars represent standard error of the mean.

The effects of training and context are depicted in Fig 5.

Because the repeated measures analysis of variance may have been too strict to reveal subtle small effects of training, and because the TR group appeared to have a slightly different response pattern compared to the NT group, we also carried out exploratory paired samples *t*-tests for each group separately to compare the ratings between the first and second session. The threshold was Bonferroni corrected for multiple comparisons and set at *p*<.006. For the TR group, we found lower arousal judgments for abstract artworks viewed in the museum (mean 3.9±0.4) compared to the laboratory session (mean 4.1±0.4) (*t*(19) = 3.153, *p* = .005). No other differences between the two sessions were found. On the other hand, both the NT group and the Expert group rated abstract artworks and portraits viewed in the museum as less liked/wanted than paintings viewed on a computer monitor in the lab (NT group—abstract artworks: *t*(19) = 3.204, *p* = .005, portraits: *t*(19) = 3.077, *p* = .006; Expert group—abstract artworks: *t*(19) = 4.439, *p*<.001, portraits: *t*(19) = 3.958, *p* = .001). It is not clear why the TR group maintained their judgement of liking while the art novices without training and the experts showed decreased evaluations of liking for artworks in the museum. One possible explanation is that the TR group received very specific information relevant to the particular artworks in the exhibition, which aided their understanding and general perception of the artwork. However, we would have expected the Expert group to also be aware of the background knowledge needed to understand the artworks in this exhibition because of their education and training. A different account may be that receiving a training prior to the start of the session may have affected participants in some way, perhaps making them feel special, or feeling a heightened responsibility to evaluate the paintings to the best of their knowledge now that time had been invested in them through the short training session. Speculatively, one could argue that maintaining a level of perceived liking (compared to a decrease in evaluations) suggests that the TR group may have enjoyed viewing the artworks more. However, we did not directly measure this, and further research is needed to better understand how people can be best provided with information in order to make the most of an art exhibition or museum visit.

Overall, the above analysis did confirm differences in ratings between art experts and novices on the more cognitively-influenced aspects of art viewing and judging. Unexpectedly, we found that participants seemed to prefer artworks viewed in digital format in the laboratory setting over viewing them in full glory in the museum, rating the artworks seen in the lab as more beautiful and more liked personally.

### Effects of familiarity

In their second session at the museum, participants rated 150 artworks, half of which had been previously seen in the first session. It was therefore possible to test for effects of familiarity. Ratings from the museum session were entered into a 3 by 2 by 2 repeated measures analysis of variance with Group (NT, TR, Expert) as a between-subjects factor, and Familiarity (Familiar, New) and Art Type (Abstract, Portrait) as within-subject factors.

#### Valence

We found no significant interactions or main effects for valence ratings of familiar and novel artworks seen in the museum.

#### Arousal

Arousal scores showed a significant main effect of Art Type (*F*(1,57) = 57.936, *p*<.001, η = .504), indicating that in general, participants rated portraits as calmer / less arousing (mean 3.4±0.8) than abstract artworks (mean 4.0±0.7). No other main effects or interactions were found.

#### Beauty

The analysis revealed a significant main effect of Art Type (*F*(1,57) = 10.561, *p* = .002, η = .156) and Familiarity (*F*(1,57) = 12.464, *p* = .001, η = .179). Portraits were rated as more beautiful (mean 4.0±0.8) than abstract artworks (mean 3.7±0.8). Familiar artworks—regardless of Art Type—were deemed more beautiful (mean 3.9±0.7) than artworks that were viewed for the first time (mean 3.8±0.7).

#### Liking

There was a significant main effect of Familiarity (*F*(1,57) = 22.381, *p*<.001, η = .282). Artworks that had been seen in the previous session were rated as higher (mean 3.4±0.9) than artworks that were viewed for the first time (mean 3.2±0.8).

The absence of any group effects with regard to familiarity was somewhat surprising as we expected mere exposure effects to be modulated by levels of art expertise. We carried out exploratory paired samples *t*-tests for each group separately to investigate the lack of group differences further. For each group, we compared evaluations of beauty and liking for both abstract artworks and portraits that had been viewed in the baseline session (‘familiar’) or not (‘novel’). We applied a Bonferroni correction to correct for multiple comparisons and set the threshold for statistical significance at *p*<.00625. Results showed that the NT group did not show any difference in evaluations for familiar versus unfamiliar artworks (*t*(19)<2.289, *p*>.034 for all). The TR group found familiar portraits more beautiful and more likable compared to the novel portraits (beauty–*t*(19) = 4.026, *p* = .001; liking–*t*(19) = 3.093, *p* = .006) but did not show any familiarity effects for abstract artworks (beauty–*t*(19) = 1.271, *p* = .100; liking—*t*(19) = 1.728). The Expert group, on the other hand, again showed no difference in their ratings for artworks previously seen in the baseline session compared to paintings that were seen during the museum session only (*t*(19)<2.067, *p*>.053 for all). Of note, it is possible that participants in the Expert group were generally more familiar with the artistic styles used in our stimulus set. Although no participant had visited this particular exhibition before, we cannot exclude that some of the experts may have come across the artworks previously.

**Fig 6:**
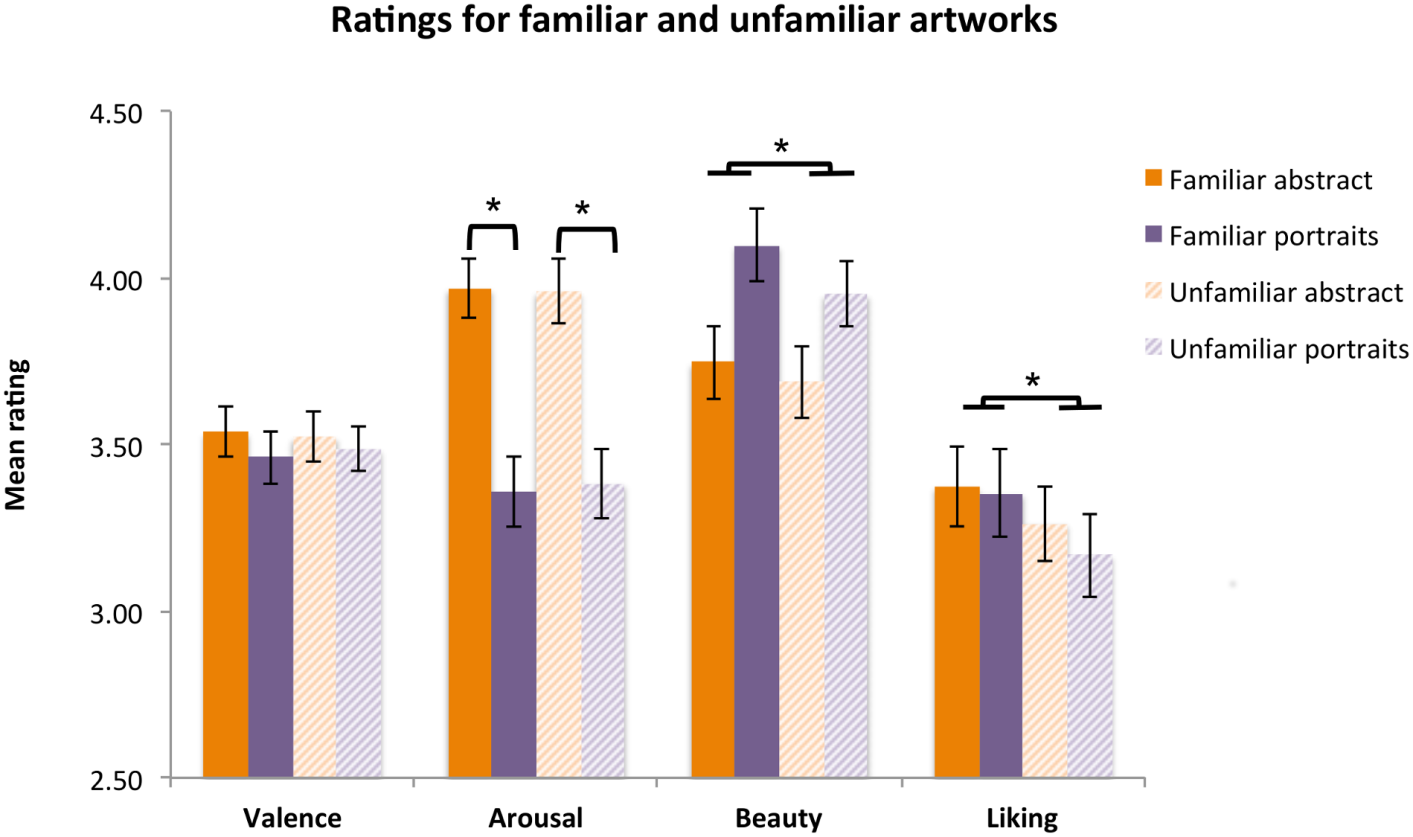
Effects of familiarity on rating scores obtained in the museum session. Error bars represent the standard error of the mean. * significantly different from each other at *p*<.05.

To summarise, as shown in Fig 6, we observed mere exposure effects only for the more cognitively-influenced aspects of art viewing (Beauty and Liking ratings). Paintings that had been previously seen were rated more beautiful and more liked personally than those that had not been viewed before. Familiarity effects occurred for both portraits and abstract artworks.

## Discussion

The current study investigated the role of art expertise with respect to basic affective judgments and more cognitively modulated evaluations of artworks. We also compared the effect of viewing environment (laboratory versus museum), and effects of familiarity with the artworks. We included a large number of artworks (150 in total), both representational (portraits) and abstract.

Our study found that art experts rated both types of artworks as more beautiful and more preferred compared to art novices. Experts and novices did not differ in affective evaluations of the artworks (valence and arousal). Unexpectedly, we found that observers rated the artworks viewed in the laboratory on a computer monitor as more beautiful and more likable. However, naïve observers who received a brief training in art history and background did not show this effect: they found the artworks in the museum just as likable as the ones viewed in the laboratory session. In the museum session, all participants—irrespective of expertise level—preferred familiar artworks (seen previously in the laboratory session) compared to artworks that they had not seen before. We will discuss each finding in more detail below.

### The role of art expertise

In the present study, we found that there were differences between art experts and novices on cognition-oriented judgments of beauty and liking, while affective judgments on valence and arousal were comparable between these two groups. We observed a positive correlation between level of art expertise and the cognition-oriented beauty / liking ratings, while no such correlation existed for the affective valence / arousal ratings. This finding supports the idea that these aesthetic judgments are mediated by the observers’ knowledge and expectations regarding a particular artwork [7,30].

In contrast, the consistency in affective evaluations across observers substantiates earlier reports by our group [39,40]. Our previous results suggested that at least some part of the emotional response to artworks (both abstract and representational) operates independently of art expertise and is reliant on visual characteristics of an artwork. These finding have implications for claims regarding the universality of art, given the ability of many artworks to appeal to viewers from different cultures and time periods [58]. Our results support the idea that art expertise plays an important role in art appreciation in general, in particular for judgments of beauty or importance of an artwork, but that some basic, emotional component of experiencing art may be universal and is thus comparable across art experts and novices alike. The current results show an interesting interaction between the role of visual aspects of the artwork and factors pertaining to context and expertise, supporting the notion that the perception of art is a complex phenomenon.

### Effects of training

It was interesting to find that even a brief training manipulation had a small influence on the ratings. The non-training group and the expert group all rated the paintings viewed in the museum as significantly less preferred compared to the lab session, but this effect was absent in the training group, who rated paintings across the two sessions as equally preferred.

Unexpectedly, the training group also rated the abstract artworks as significantly less arousing following the training. One explanation may be that perhaps the increase in background knowledge following the training somehow made the painting less novel and therefore less exciting. However, our data with regards to familiarity show no difference in arousal ratings for novel versus familiar paintings. In addition, the expert group also shows lower arousal ratings in the museum session (albeit for portraits), although the level of background knowledge remained unaltered in this group. Thus, although a simple 30-minute lesson on the artists and art history related to an exhibition was sufficient to alter the degree to which viewers liked artworks, the effects of the training were not clear-cut and need to be further investigated in future studies, perhaps by testing different types and durations of training.

### Museum / lab context

We were also interested to see whether artworks would be better appreciated in a museum, which is the culturally defined location where artworks are supposed to be best viewed (as opposed to a dimly lit lab room). Surprisingly, we found the opposite trend, in particular for evaluations of liking. Our participants were much less likely to report wanting to hang a painting on the wall at home after seeing it in the museum. There are several possible explanations for this unexpected result. First, it is important to note that the artworks in this particular exhibition were shown in relatively large groups, rather than more isolated as is sometimes the case. This might have made it more difficult to enjoy the artwork, or the artwork might have seemed less interesting compared to other works that participants saw nearby. Also, the exhibition itself was very large and participants often took several hours to complete their visit. Hence, mental or physical fatigue may have played a role, consistent with studies of ‘museum fatigue’ (for a review, see [59]).

### Effects of familiarity

We found that participants rated repeated stimuli (those seen in both the lab and the museum session) as more beautiful and likable compared to artworks that were only seen in the museum session. This is in line with suggestions that the mere exposure effect [50] applies also to artworks [10].

Contrary to our expectations, we found no main effects or interactions with respect to art expertise. Exploratory analyses showed that within each group, only the participants who had received a brief training rated portraits but not abstract artworks that they had seen in a previous session as more beautiful and more preferred.

The fact that we observed familiarity effects only on the more cognitively-mediated aspects of art evaluation suggests that having encountered an artwork before helps observers to appreciate its artificial merit and significance. With respect to providing specific art historic and stylistic information, we found some evidence that this helps art novices to appreciate representational artworks that they had encountered on a previous occasion. These findings are directly relevant to museums, as they suggest that promoting artworks in an exhibition (for example by providing a preview of the artworks in the exhibition online, along with brief background information) may increase the overall experience of non-expert museum visitors.

### Conclusion

In sum, the overall pattern of results is consistent with two major trends. First, basic affective evaluations of both abstract and representational artworks were comparable across groups with different levels of art expertise. This is consistent with the theory that the perception of emotional expression in artworks can be highly consistent across observers, perhaps due to the fact that visual features such as colour and shape can have an affective value [39,40]. The ability of certain artworks to evoke a response across time and different cultures may help to explain why some artworks achieve a special status as great masterpieces [35,58]. Second, an important aspect of beauty (and preference) does lie in the eye of the beholder. Ratings of beauty and preference/liking were influenced by the level of art expertise, and by the context in which the image/artwork was viewed. This is consistent with theories emphasizing a cognitive influence on the perception of art [15,21].
